# Point-of-Use Rapid Detection of SARS-CoV-2: Nanotechnology-Enabled Solutions for the COVID-19 Pandemic

**DOI:** 10.3390/ijms21145126

**Published:** 2020-07-20

**Authors:** Navid Rabiee, Mojtaba Bagherzadeh, Amir Ghasemi, Hossein Zare, Sepideh Ahmadi, Yousef Fatahi, Rassoul Dinarvand, Mohammad Rabiee, Seeram Ramakrishna, Mohammadreza Shokouhimehr, Rajender S. Varma

**Affiliations:** 1 Department of Chemistry, Sharif University of Technology, Tehran 11155-3516, Iran; nrabiee94@gmail.com (N.R.); bagherzadeh@sharif.edu (M.B.); 2 Department of Materials Science and Engineering, Sharif University of Technology, Tehran 11155-9466, Iran; ghasemi.mse@gmail.com; 3 Biomaterials Group, School of Materials Science and Engineering, Iran University of Science and Technology, Tehran 16844, Iran; zare.hossein.1989@gmail.com; 4 Student Research Committee, Department of Medical Biotechnology, School of Advanced Technologies in Medicine, Shahid Beheshti University of Medical Sciences, Tehran 19857-17443, Iran; speahmadi@yahoo.com; 5 Cellular and Molecular Biology Research Center, Shahid Beheshti University of Medical Sciences, Tehran 19857-17443, Iran; 6 Department of Pharmaceutical Nanotechnology, Faculty of Pharmacy, Tehran University of Medical Sciences, Tehran 14155-6451, Iran; youseffatahi@gmail.com (Y.F.); dinarvand@tums.ac.ir (R.D.); 7 Nanotechnology Research Center, Faculty of Pharmacy, Tehran University of Medical Sciences, Tehran 14155-6451, Iran; 8 Universal Scientific Education and Research Network (USERN), Tehran 15875-4413, Iran; 9 Biomaterial Group, Department of Biomedical Engineering, Amirkabir University of Technology, Tehran 15875-4413, Iran; mrabiee@aut.ac.ir; 10 Center for Nanofibers and Nanotechnology, National University of Singapore, Singapore 117576, Singapore; seeram@nus.edu.sg; 11 Department of Materials Science and Engineering, Research Institute of Advanced Materials, Seoul National University, Seoul 08826, Korea; 12 Regional Center of Advanced Technologies and Materials, Palacky University, Šlechtitelů 27, 78371 Olomouc, Czech Republic

**Keywords:** SARS-CoV-2, nanoparticles, nanotechnology, point-of-use, rapid detection of virus, COVID-19

## Abstract

Severe acute respiratory syndrome coronavirus 2 (SARS-CoV-2) caused the COVID-19 pandemic that has been spreading around the world since December 2019. More than 10 million affected cases and more than half a million deaths have been reported so far, while no vaccine is yet available as a treatment. Considering the global healthcare urgency, several techniques, including whole genome sequencing and computed tomography imaging have been employed for diagnosing infected people. Considerable efforts are also directed at detecting and preventing different modes of community transmission. Among them is the rapid detection of virus presence on different surfaces with which people may come in contact. Detection based on non-contact optical techniques is very helpful in managing the spread of the virus, and to aid in the disinfection of surfaces. Nanomaterial-based methods are proven suitable for rapid detection. Given the immense need for science led innovative solutions, this manuscript critically reviews recent literature to specifically illustrate nano-engineered effective and rapid solutions. In addition, all the different techniques are critically analyzed, compared, and contrasted to identify the most promising methods. Moreover, promising research ideas for high accuracy of detection in trace concentrations, via color change and light-sensitive nanostructures, to assist fingerprint techniques (to identify the virus at the contact surface of the gas and solid phase) are also presented.

## 1. Introduction

Since the novel coronavirus disease 2019 (COVID-19) appeared for the first time in Hubei Province, China, in December 2019, cases of the virus have been reported in almost every country in the world within three intervening months [1,2]. It has been termed a smart virus because it—like its counterparts (such as Ebola) [3,4,5]—can show different reactions and symptoms in living organisms, and can even optimize these reactions and symptoms by increasing the proliferation of its genetic material for survival. The well-known symptoms of the virus often start from mild fever and shortness of breath, followed by severe cough and failure of the immune system [6,7]. The initial diagnosis of the virus is critical and vital for the community. In this context, scanning of the patient’s lungs using computed tomography (CT) has assisted with possible analysis of the varied range of opacities as the handy test in the clinical phases. However, this technique can only reveal the first assessment of the patient’s lungs and cannot be solely used for further therapies. Further analysis is essential to confirm the level of the virus in the patient’s body, including a multiplex real-time polymerase chain reaction (RT-PCR) that is considered as the negative control results group [8,9,10]. Based on recent studies, by January 2020, this virus has similar genetic sequence materials to the betacoronavirus B lineage, with some differences. Furthermore, the discovered pathogen has been compared to other known viruses and syndromes, wherein it has ~50% similarity to Middle East respiratory syndrome virus (MERS-CoV), ~80% similarity to severe acute respiratory syndrome virus (SARS-CoV), and ~96% similarity to bat coronavirus RaTG13 [11,12]. To date (14 July 2020), more than 13 million people have been infected, and more than 500,000 have died as a result of the unprecedented growth of the virus worldwide. These statistics prompt an urgent need for increased research in this field so that identification by the most accurate diagnostic methods, as well as epidemic treatments, can be undertaken at the earliest [13,14,15].

SARS-CoV-2 is transmittable from human to human at the rate of more than three people per a confirmed infected person; the virus can spread dramatically in the globalized world, and, apparently, no detection method is available in clinical facilities that can inhibit that. Therefore, the necessity of home-based and point-of-use diagnosis techniques are highly desirable at this juncture. Since the virus, in most cases, does not show very acute symptoms, and the similarity of these indications to viruses, such as influenza, is rather high, these indicators cannot be emphasized enough for home-based diagnosis techniques. However, there is a characteristic of this virus that could help scientists find an optimized, home-based virus detection method. That trait is its ability to survive in aerosols with a size of less than 5 µm for more than 3 h, and its possible movement in the cloud, up to 8 m [16,17,18,19].

The search for means to prevent, diagnose, and treat the virus continues, but so far, investigations into the diagnosis and treatment of the virus have not yet reached the Food and Drug Administration (FDA) approval stage [20,21]. The current diagnosis procedure is based on symptom onset, sample collection, and infection control measures in the first step, following by collecting and transporting the sample to the laboratories, testing them, and even sequencing the samples in some cases; this procedure apparently cannot work under epidemic conditions, which could translate to global catastrophe [22,23,24].

In the meantime, scientists, besides improving expeditious prevention and diagnostics while enhancing their quality and accuracy, are striving for creative solutions; viability of the virus in the air, and size and surface morphology of the virus, need to be exploited. Based on recent studies, the diameter of the SARS-CoV-2 is ~60 to 140 nm, which includes its genetic material, as well as the envelope protein spikes on the surface; four structural proteins present will be discussed in the sections below [25,26,27,28,29,30]. Meanwhile, three-dimensional chemical structures can play an indispensable role in the uptake and identification of these types of viruses. One of them is Metal-Organic Frameworks (MOFs), which is a class of porous coordination polymers (PCPs) consisting of clusters and metal ions, along with polytopic organic linkers [31,32,33]. MOFs are porous structures with high surface areas and wide application potential. Composites comprising MOFs with other functional materials have been used in biomedical applications, such as sensors, separation, and biocatalysis. Moreover, the MOFs have useful fluorescence quenching ability toward fluorescence-labeled DNA probes [34].

**Figure 1:**
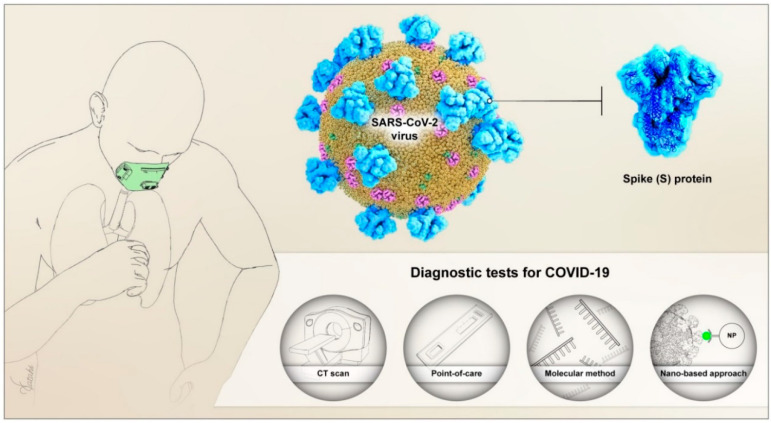
Schematic illustration of common Lung computed tomography (CT) and molecular test for diagnosis of severe acute respiratory syndrome coronavirus 2 (SARS-CoV-2), and emerging detection methods, based on the optical-based biosensor, Point-of-Care (POC) testing, and the optical-based nanosensor.

In this article, we focus on a new and fully adaptable technique based on the MOFs and analytical methods, especially colorimetry, to provide a handy, smart, cost-effective, and simple diagnosis technique for home-based prevention and diagnosis actions. For comparison purposes, all other detection methods are also illustrated in Figure 1. Building on this background, this article also proposes a new and highly sensitive technique based on nanotechnology for the detection of SARS-CoV-2 on the contact surface of gas and solid phases, including masks and clothes.

## 2. Characteristics of SARS-CoV-2 towards the Possible Mechanism of Diagnosis and Treatment

**Figure 2:**
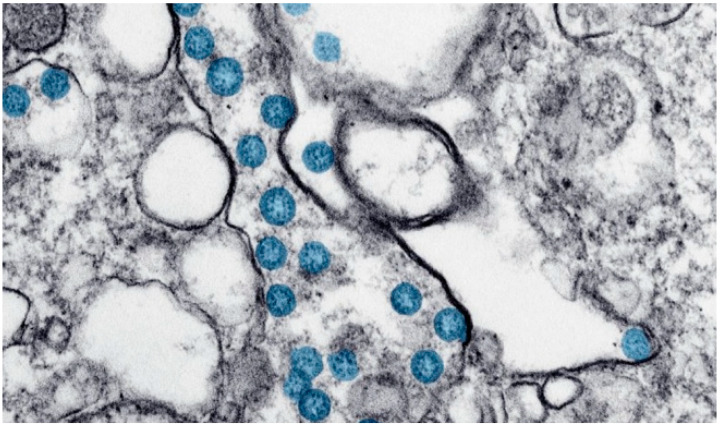
Transmission electron microscope image of SARS-CoV-2 spherical viral particles in a cell [39]. Reprinted (adapted) with permission from the American Chemical Society (2020).

The SARS-CoV-2 virus was initially discovered in a patient sample in Wuhan, China, and the related epithelial cells were cultured with the SARS-CoV-2, which have been isolated from the patients (Figure 2). The mediated supernatants were collected and analyzed based on the negative-stained transmission electron microscopy (TEM) and revealed that the diameters of the SARS-CoV-2 are up to 140 nm, which includes its genetic material, as well as envelope protein spikes [35]. Generally, the SARS-CoV-2 has included the RNA genome that consists of about 30,000 nucleotides, and the resulting genome comprises 27 proteins that have all been encoded and comprise RNA-dependent RNA polymerase (RdRP) in the main structure, including four structural proteins [36,37]. The 96% similarity of the genome of SARS-CoV-2 to RaTG13 emanates from the RdRP gene region, most of the sequence homology was observed being similar, except 0.1% of them that showed sequence diversity [38].

There are three structural proteins on the surface of SARS-CoV-2, including small envelope protein (E), spike surface glycoprotein (S), and matrix protein (M), in which the S protein is more accessible than the others in the face of external chemical structures, and media, and one structural protein inside the membrane of SARS-CoV-2, which is termed nucleocapsid protein (N). Based on the newer findings, the S protein is accountable for more than 80% interaction of the virus with the cellular membrane and is also responsible for infecting the cells through receptor-binding mechanisms. Consequently, a smart solution for prevention, early diagnosis, or even treatment would be targeting this protein via chemical, physical, or biological approaches. Generally, the structural analysis of the E and S proteins shows the chemical structure encompassing NH_2_-, -L-Cys-A-Y-Cys-Cys-N-, -COOH, NH_2_, and -S-Cys-G-S-Cys-Cys-K-, respectively [40,41,42]. In this regard, it is possible to have a strong interaction between these two motifs by disulfide bonds; therefore, the possible mechanism for destroying the structure of the SARS-CoV-2 would certainly entail targeting the disulfide bonds or interactions. From another perspective, aiming for these targets would be considered as the smart choice for designing optical sensors for the detection of SARS-CoV-2, which can be used in early diagnosis, and for the detection of viruses even on the surfaces of metals, clothes, and wood, etc.

**Scheme 1:**
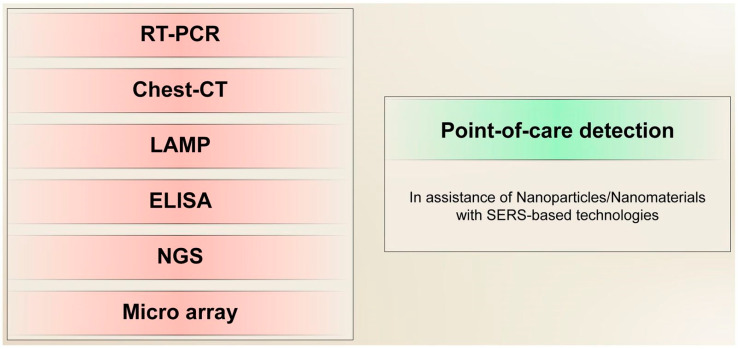
An illustration of the scope of this article for highly sensitive point-of-care detection.

Regarding the interaction of the SARS-CoV-2 with different range of cell lines, there are a few studies that revealed the virus does interact with the angiotensin-converting enzyme 2 (ACE2) [11,43,44,45]. Although the exact chemical mechanism is not defined, it appears that the S and E proteins have a critical role in this type of interaction and subsequent entry into cells; therefore, inhibition of these proteins via their degradation or even physical deactivation would be helpful in the elimination of the cellular infection mechanism. It should be noted that the ACE2 is present in different tissues and organs, including venous and arterial endothelial cells, as well as smooth muscle cells in the intestine, stomach, and lungs. Hence, the choice of the nanomaterial selected as the inhibitory agent referred to in the preceding paragraph should be considered carefully with low cytotoxicity, as well as of the appropriate size for use in these tissues. Additionally, it should be noted that if the virus is to be targeted with a nanomaterial inside living human tissue, the separation of the nanomaterial should also be considered. Therefore, it is ideal to use nanosystems that can be easily implanted in the body and removed after they have served their purpose where the use of MOFs may be perfect. It should be noted that other nanomaterials may be considered, but due to the special structural features of MOFs, we emphasize these nanostructures. These nanostructures endowed with interconnected porosity can even allow the virus to enter in optimized and large structures. Alternatively, even if they cannot enter into the virus, the surface proteins (the possible protein being S protein) can physically (in the simple state) or chemically (in the modified state) be adsorbed or absorbed on the surface of the entering channels of the MOFs. In addition, the high ratio of surface to volume of MOFs makes them promising candidates for adsorption of different sub-micron sized pathogens. Consequently, it is possible to detect the exact concentration of the pathogen by adsorbing the pathogens in MOFs and utilizing different physiochemical detection methods. In addition, the ultra-fast detections can be achieved using the absorption routes, the optical mechanisms, based on Off–On or On–Off, and quenching approaches. The main scope of this article, appraising the past, and common methods towards point-of-care detection methods, is illustrated in Scheme 1.

## 3. Current Diagnostic Techniques for the Detection of SARS-CoV-2

**Figure 3:**
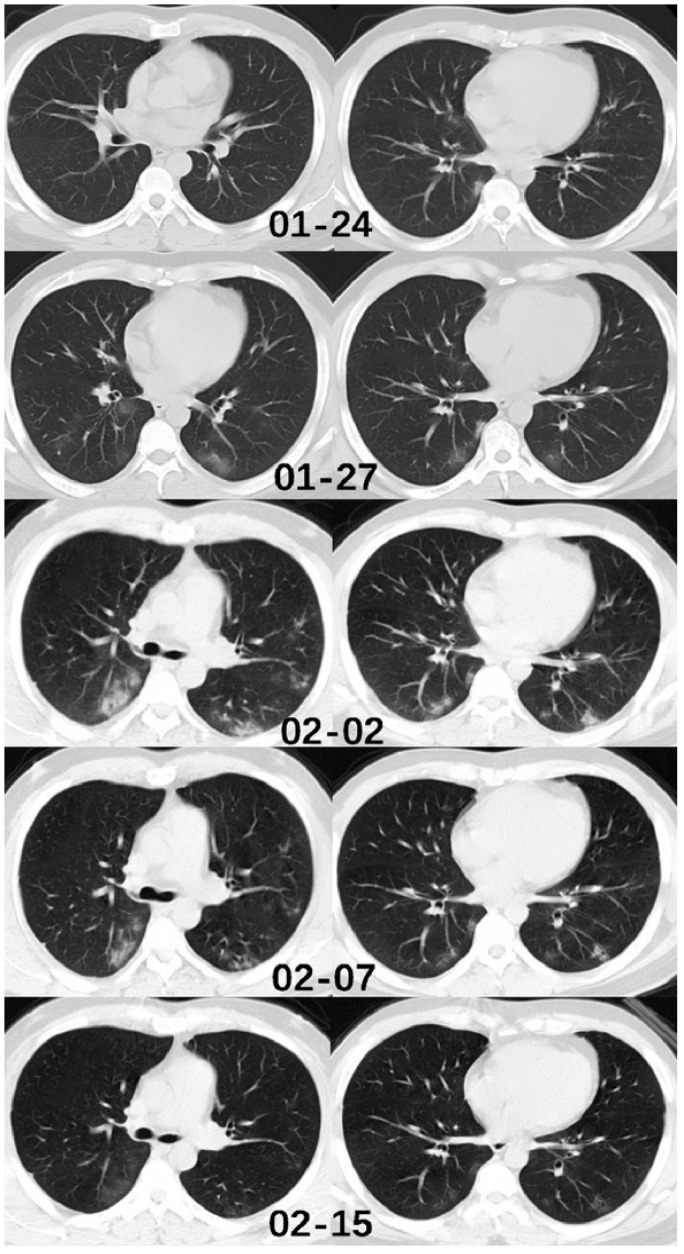
Chest CT of a 37-year-old male patient. This individual returned from Wuhan to Wenzhou on January 19 and inflicted with cough and expectoration. The first chest CT was conducted on January 24, which showed subtle peripheral ground-glass opacity in the middle lobe, and right inferior lobe. The second CT images, of January 27, and the third examination, on February 2, showed a significant increase of lesion numbers and density, especially in both lower lobes. The fourth CT images, of February 7, showed a decrease in the density of the pulmonary lesions. On the fifth CT examination of February 15, the lesions were absorbed, and the patient was discharged [52]. Reprinted (adapted) with permission from Elsevier.

Many methods and kits have been reported in the literature to detect SARS-CoV-2 after isolation from patients and identification of its sequence with China’s whole genome sequencing technique. RT-PCR, chest CT imaging is the first and most common diagnostic techniques in the detection of COVID-19 (Figure 3). RT-PCR is the gold standard in the detection of COVID-19, which is based on the synthesis of cDNA from genomic RNA and is followed by amplification [46]. The major problem of RT-PCR is its low sensitivity to chest scans due to the insufficient number of viruses in the blood or the inaccuracy of the laboratory kit [47,48]. Chest-CT can be used to identify patients who need more testing, isolation, and treatment, and is more sensitive than RT-PCR; however, it is not entirely specific for COVID-19 and has been shown to diagnose other diseases with symptoms similar to corona [8,49,50,51].

With the high prevalence of COVID-19, many companies, such as ScienCell [53], Thermo Fisher Scientific [54], and Qiagen [46], etc., have developed commercial quantitative real-time PCR (RT-qPCR) diagnostic kits for the SARS-CoV-2 epidemic. RT-qPCR testing by specific primers design in *NP* and *ORF1ab* genes have been developed to detect the virus in samples [54]. Despite its widespread use, the need for expensive equipment, more time duration, and the need for high purity samples are some of the limitations of this technique. Due to the variability of the number of viruses in different samples in patients, false negative results are also obtained [22,55].

Loop-mediated isothermal amplification (LAMP) technique is a nucleic acids amplification assay that has been studied widely by many researchers. The emergence of this method is due to its specific primers, a Bst DNA polymerase enzyme with chain displacement activity under isothermal conditions, without needing thermocycler or electrophoresis equipment [56,57]. The high sensitivity and rapidity of this method have made it an appropriate choice for the detection of the various virus such as MERS-COV [58], SARS-COV [59], and influenza A [60]. LAMP techniques are very sensitive, specific, and faster than conventional PCR methods. [61]. A combination of reverse transcription and LAMP (RT-LAMP) provided a one-step high-throughput detection method for genomic RNA of SARS-CoV-2, with 100 copies/reaction of an RNA virus. The detection time of this method is 30 min, and it could be applied for POC tests and screening tests. This assay is much simpler and faster than RT-qPCR and does not need complex equipment [62].

Several immunoassay methods have been developed for the diagnosis of serum antibodies and viral proteins (N and S) of SARS-CoV-2. Most commercial kits use enzyme-linked immunosorbent (ELISA), rapid lateral flow immunoassay (LFIA) assays, as well as IgM and IgG detection from the second week of viral infection [63]. There are various companies in the field of immunoassay and rapid IgM/IgG tests for the diagnosis of COVID-19. The test from BioMedomics Co, Massachusetts, USA, is based on POC lateral flow (LF) immunoassays systems and capable of measuring IgG and IgM from plasma and serum within 10 min, and requires only very small amounts of samples [64]. Chembio, NY, USA, recently launched a Dual Path Platform (DPP) COVID-19 IgM/IgG test, which offers results in 15 min using a finger-pricked blood sample [65]. Since IgM and IgG are detectable in two weeks after the onset of infection, antibody-based diagnosis is, therefore, usually possible in the recovery phase; thus, requiring the use of other diagnostic assays for faster detection [66]. Table 1 summarizes presently available methods with their advantages and limitations in the detection of COVID-19.

### 3.1. Isothermal Nucleic Acid Amplification

The loop-mediated isothermal amplification (LAMP) method entails the amplification of DNA and RNA molecules, and is a technique with high efficiency, specificity, and rapidity. This technique uses four specially designed primers and a DNA polymerase enzyme with strand displacement activity to mark six sequences on targeted genetic material [71]. If the LAMP is combined with reverse transcription, it can be helpful in the amplification of RNA molecules [72]. During the severe acute respiratory syndrome (SARS) epidemic era, a LAMP-based approach was applied, and amplified products that were analyzed with gel electrophoresis. These earlier comparative studies concluded that the findings with those of the conventional PCR-based technique are more accurate than the LAMP-based approach as the latter is not a quantitative technique to identify SARS-CoV RNA [73]. In a similar study, the final LAMP primers were chosen from the Replicase 1b region in SARS-CoV RNA to perform an RT-LAMP system under 63 °C isothermal conditions. As the reaction continues, pyrophosphate and magnesium ions bind to each other to form magnesium pyrophosphate, and the progress of this reaction can be measured with a turbidimeter [74]. This quantitative approach reduced the limitations of endpoint detection in the LAMP technique, which usually is viewed by applying electrophoresis [75]. Based on this concept, a high specificity RT-LAMP test was suggested for the surveillance of Middle East Respiratory Syndrome coronavirus (MERS-CoV) as the reaction could detect four genome copies of MERS-CoV in less than 60 min. This study compared two different fluorescent dyes and concluded that EvaGreen has higher signal read-out properties than SYBR Green. However, this approach is not succeeding as a promising method for early diagnosis and detection of COVID-19, due to the limitations in the sensitivity, as well as the necessity of high loading of the virus.

Furthermore, field-deployable microchambers lead to the identification of 0.4 genome copies, among other acute respiratory disease viruses, such as H1N1 and H3N2 [76]. There is also another report that focused on the detection of novel swine acute diarrhea syndrome coronavirus (SADS-CoV) by using the N gene of the virus, and the study reported its limit of detection equals to 10 copies/μL without observing any cross-reaction among other swine fever viruses [77]. Therefore, the aforementioned limitations are still prevailing in this technique. An advantage of RT-LAMP compared to conventional RT-PCR is its higher sensitivity, but it is not adequate for the surface detections based on a very low amount of virus. Furthermore, RT-LAMP does not lose any time for a thermal change, so it is a more efficient approach compared to RT-PCR. As affirmed in a study by Thai et al., RT-LAMP is 100-fold more sensitive than RT-PCR in SARS-CoV surveillance with specificity being 100% and 87%, respectively. Finally, using a turbidimeter renders RT-LAMP a real-time approach [78]. However, this technique still can only be deliberated for the detection of the virus in the patients’ having a considerable load of the virus and is not applicable for the early detection of viruses on different surfaces and coatings.

The analysis of results from the LAMP reaction usually is through gel electrophoresis or a turbidimeter (an instrument can quantitatively measure the intensity loss of light scattered from the suspended particles) [79,80]. However, another disadvantage of conventional LAMP-assay lies in the ambiguity of selecting the correct signals from the noises caused by other sources. Such noises can be related to solution turbidity because of the release of the pyrophosphate during polymerization, fluorescence dyes intercalation into any dsDNA amplicons, and false signals from non-primer reactions [79].

As an example, a study engaged quenching probes in a fluorescent RT-LAMP assay to detect MERS-CoV. Quenching probes could solve the problem of solution turbidity during monitoring the process because a positive signal in this method just relates to the primer-reaction [80]. Additionally, a colorimetric approach designed by Huang et al. to produce two specific sets of inner primers, where one had a TTTT sequence and the other did not, to conduct a combinatory system between RT-LAMP and a vertical flow visualization strip for identifying N gene of MERS-CoV. The advantage of this technique is that the final results could be seen by the naked eye just after 5 min with a 10-fold higher limit of detection than the conventional RT-LAMP system, which is the right approach for detection of the virus on different surfaces. Two loop primers participating in this isothermal amplification process are labeled with fluorescein isothiocyanate (FITC) and biotin. So, the amplicons are labeled with both FITC and biotin after amplification. Those labeled with biotin had an affinity to bind gold nanoparticles (NPs) conjugated on streptavidin complex and the other amplicons labeled with FITC captured by an anti-FITC antibody. These antibodies were immobilized on the text line of the strip [79]. This approach can be contemplated as a model study for the detection of a low amount of the virus on the masks and even on clothes, but the most cost-effective and simple systems can be designed as well. Another suggestion to address the intercalating dyes usually used in real-time LAMP fluorescence detection is to utilize a toehold mediated strand exchange reaction, called one-step strand displacement (OSD). Since the OSD is a sequence-specific reaction, it can successfully separate false signals from the correct signals [81]. Likewise, a study employed OSD to identify LAMP amplicons in a sequence-specific system, and it could successfully sense 0.02 to 0.2 PFU of MERS-CoV in infected cell culture supernatants [82]. However, this technique is still not applicable to simple masks and clothes, as it needs specific laboratory equipment, which enhances the chance of infecting healthy people.

Rolling circle amplification is another enzymatic isothermal approach to detect the viruses. The final product of rolling circle amplification (RCA) is a concatemer, including hundreds of tandem repeats, which are complementary to the circular template. Since the RCA specifically amplifies the probe signal, it has higher sensitivity than PCR-based methods. PCR tests have the risk of amplicon cross-contamination because of the exponential amplification process, while RCA can be performed with minimal reagents and under isothermal conditions [83]. Based on RCA, a study suggested an efficient assay to detect SARS-CoV for both the liquid and solid phases [84]. Recently, a digital RCA-based assay was employed to detect the Ebola virus (EBOV) using a set of padlock probes that could separately target the viral RNA and complementary RNA of all seven EBOV genes. This pump-free microfluidic chip-enabled the digital analysis without any need for specialized equipment like PCR [85].

### 3.2. Microarray-Based Technologies

Microarray assay is a microfluidic approach that can rapidly detect the virus RNA. After reverse transcription, the viral RNA produces cDNAs labeled with a specific probe to load in each well, followed by hybridization between oligonucleotides fixed on the microarray. Finally, free DNAs are removed, and the virus detected through specific probes [79]. Recently, a study introduced an antigen microarray technology using coronavirus antigens taken from epidemic coronavirus, including SARS-CoV-2 and the other family of human coronaviruses. These findings suggest that high IgG for common human coronaviruses but low IgG seroreactivity for SARS-CoV2 meant that this could be a distinctive indicator of SARS-CoV-2 from the other human coronaviruses [86]. Another study tried to produce a general DNA microarray platform to identify novel viruses that showed that novel pathogenic viruses could be detected by cross-hybridization to highly conserved sequence motifs [87]. In addition, an inexpensive oligonucleotide microarray, which depends on a non-fluorescent detection of the whole coronavirus genus after RT-PCR, enabled the limit of detection of 15.7 copies per reaction [88]. Moreover, a DNA microarray method was developed to assess 27 single nucleotide polymorphism (SNP) mutations among the (S) gene of the SARS [89]. A chip-based assay reported for the identification of SARS-CoV, which had some advantages compared to the conventional single round real-time RT-PCR including higher sensitivity (10 copies of SARV-CoV) and higher specificity. Since this method relies on three virus-specific and three control probes, it has a higher specificity and diminishes the false positive and false negative results; they used fluorescent dyes as the probe, then scanning the fluorescence intensity with a confocal scanner [90].

### 3.3. CRISPR Technology

Prokaryote cells remember an adaptive immunity memory by keeping several genetic elements of pathogenic agents in genomic loci called CRISPR arrays. So the application of CRISPR-Cas biology can help assist in the early diagnosis of infectious agents [91]. Based on CRISPR-Cas enzymology, a group of researchers developed a platform named SHERLOCK (specific high sensitivity enzymatic reporter unlocking), which can distinguish between various inputs that differ only a single nucleotide at very low concentrations [92]. Later, they improved this platform for Cas-13, Cas12a, and Csm6 to detect Zika or Dengue viruses [93]. Recently, Mammoth Biosciences claimed that the Endonuclease-Targeted CRISPR Trans Reporter (DETECTR) platform could rapidly detect SARS-CoV-2 in less than 30 min by applying a lateral flow strip relying on a CRISPR detection enzyme assay. Based on the published protocol, the process will have three steps: RT-LAMP in 62 C for 20 min, using Cas 12 in 37 C, which takes 10 min to distinguish the SARS-Cov-2, and finally, RT lateral flow for 2 min. The limit of detection reported being 70-300 copies/μL input [94,95]. Likewise, Liu group reported an all-in-one Dual CRISPR-Cas 12 to detect SARS-CoV-2 and human immunodeficiency virus (HIV), simultaneously. This assay uses a pair of crRNA to initiate dual CRISPR-Cas 12a detection [96]. In addition, a CRISPR-Cas13-based strategy has been implicated in destroying SARS-CoV-2 sequences in human lung epithelial cells, where a mixture of six crRNAs degrade up to 90% of all coronaviruses [97]. Furthermore, Alejandra group reported that CRISPR-Cas 12 based diagnostic tool is capable of detecting the synthetic SARS-CoV-2 RNA sequences, which is an inexpensive (1–2 USD/reaction) assay with the ten copies/μL limit of detection [98]. In a similar work, Hou et al. used Cas 13 protein to detect SARS-CoV-2 RNAs [99].

### 3.4. Point-of-Care Detection of SARS-CoV-2

There is a wide range of difference in the symptoms reported in patients, in a typical study, 44% of the patients in China had a fever before being under any therapies, but over 80% of them had developed trends in their fever after starting drug therapies. In that study, about 20% of the patients had shortness of breath; also, 68% of the patients had cough [100]; therefore, it cannot be a sanctioned method to use the first symptoms for the detection and diagnosis the SARS-CoV-2. In addition, molecular techniques have been used widely along with CT as the most accurate and sensitive technique in the diagnosis of the virus, however, the need to send samples to specific laboratories and the likelihood of healthy individuals becoming ill during the process can lead to the abolition of the method shortly afterward. On the other hand, prevention is always better than cure, so the need for research advances in technologies that lead to virus prevention is crucial. One of the simple, fast, and reliable technique for detection of the virus for the purpose of prevention is certainly based on the optical biosensors.

There are some approaches for point-of-care testing of the SARS-CoV-2 and related viruses, wherein most of them are based on the detection based on the lateral flow antigens. However, the advancements in this field are hard to achieve and optimize; therefore, using a different approach is highly suggested. However, for clarification purposes, generally, in the lateral flow antigens-based techniques, there is a paper-like membrane strip that has been chemically coated with metallic NPs conjugated with antibodies that also capture antibodies. The metallic NPs generally possess the active optical features, including Surface-Enhanced Raman Scattering (SERS) or Chelation Enhanced Fluorescence (CHEF) or other types of optical and fluorescence activity. Consequently, in commercial point-of-care testing methods that are based on lateral flow antigens, gold (Au) NPs have been used widely [101,102,103,104]. In this method, the patient’s sample, urine, or blood, depending on the nanosystem, is placed on the synthesized and modified membrane, and by using capillary action, the targeted proteins drawn across the modified strip. The mechanism is based on binding the antigens to the Au NPs by passing through the first line of the membrane. By this method, sharp and obvious color changes (that depend on the size, shape, and aggregation of those NPs) have been made, which is an indicator of the interaction with the virus. This method has achieved acceptable clinical sensitivity, accuracy, and specificity for IgM and IgG [79,105], but the need for sample collection, even urine or blood, is considered as the major drawback for the use in the house as point-of-care nanosystem.

**Figure 4:**
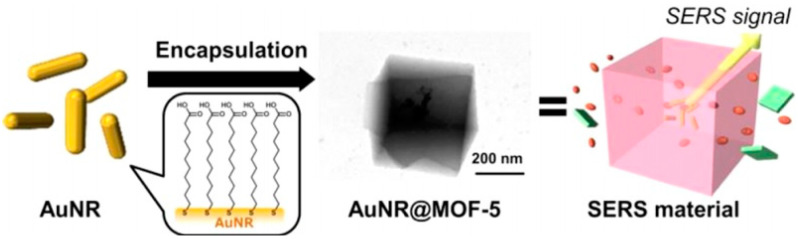
Schematic representation of Au-nanorodes@MOF-5 preparation and SERS activity. Reprinted from [106]. Copyright (2020) American Chemical Society.

Herein, we want to provide practical ideas for the design and preparation of a highly sensitive coupled with higher accuracy systems for the point-of-care detection of SARS-CoV-2 even before infection. This method is based on using MOFs, which have been synthesized via a size-selective approach. A typical synthesis entails MOF-5@Au-nanorods (Figure 4), which have been generated by Sugikawa et al. [106]. They illustrated that these types of sensors could be achieved very high accuracy as well as considerable sensitivity towards different analytes. In this study, the Au-nanorods displayed different SERS activity in the presence of pyridine derivatives. It should be noted that SERS is considered as the most effective and powerful spectroscopy technique based on vibrational frequencies, which allows the detection at very low concentration of an analyte in range of sub-attomolar quantities; the Raman scattering enhancements in range of million-folds in the procedure of adsorption of analytes on the gold NPs [107,108,109].

**Figure 5:**
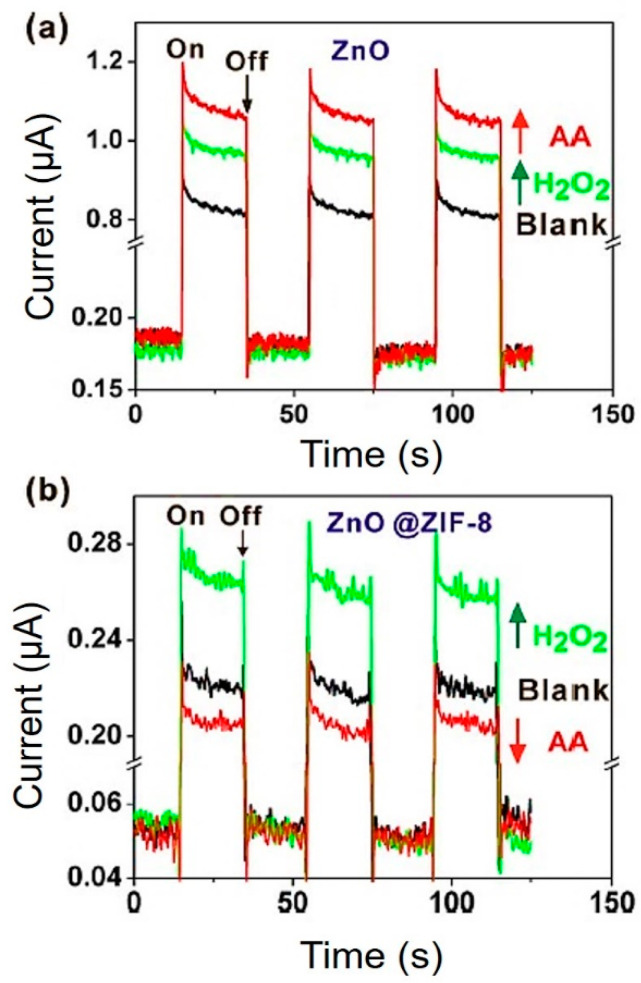
Photocurrent response of the ZnO nanorods (**a**) and ZnO-nanorodes@ZIF-8 (**b**) in the presence of H_2_O_2_ (0.1 mM). Reprinted from [110]. Copyright (2020), American Chemical Society.

Zhan et al. [110] showed that the zeolitic imidazolate frameworks-8 (ZIF-8) could be successfully fabricated with the ZnO nanorods in core-shell heterostructure chemistry which displays distinctive SERS response in sensing different molecular scavengers including ascorbic acid and H_2_O_2_ (Figure 5). The exact mechanisms came from the photoelectrochemical response of the as-prepared nanostructure to the hole scavengers. This method offers the promise that one can use these types of nanostructures to detect and diagnose viruses with a high limit of detection as well as sensitivity in the presence of different ranges of hole scavengers. This technique can be used for the early diagnosis and detection of different pathogens. For this purpose, different types of optically active components can be incorporated into the MOFs’ structure or impregnated on the surface of them. By conjugating a stimuli-responsive linker on the surface of the MOFs, an optical mechanism based on Off–On or On–Off biosensor can be proceeded [111,112,113].

**Figure 6:**
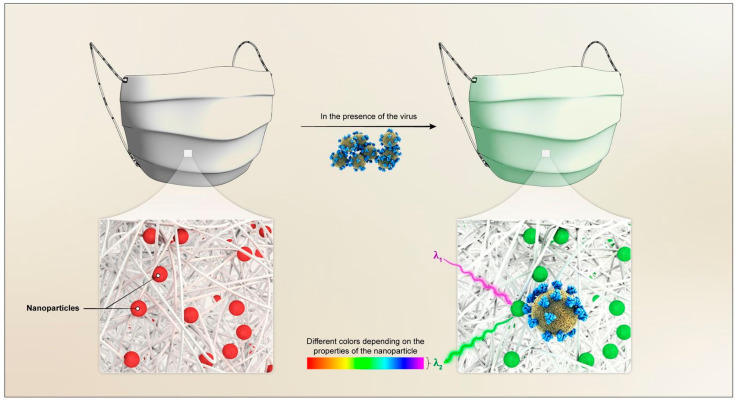
Schematic illustration of SERS intensity and color changes of mask-embedded nanoparticles (NPs) in the present of SARS-CoV-2.

Our proposal for the detection and diagnosis of SARS-CoV-2 raises an important question about the detection capability based on SERS mechanism in the presence of different hole scavengers and, eventually, their origin in the detection and diagnosis of the virus. There is a possible rationale to offer hole scavengers in the mentioned nanosystem, when the metal is present, for example, Au NPs interacting with disulfide bonds in the above examples, and disruption of this interaction. In the interim, ozone in the pores of the MOF with a sensitive capping agent may be released when gold interacts with the virus’s surface proteins. The cleavage of the cysteine bonds on the virus’s surface proteins would lead to eliminating and destroying the SARS-CoV-2’s membrane, followed by releasing the genetic material along with the virus media. In this regard, the interactions between the eliminated protein structures and the genetic material with the Au NPs would be akin to the electron-hole effect, wherein the SERS intensity would be different in this case, and also the color of the Au NPs apparently would change (Figure 6). It should be noted that the detection of different pathogens and viruses does not require their entry into the porosity of nanomaterial. In this technique, by doping or impregnating active optical nanoparticles, the virus or pathogen can be identified by establishing even weak physical interactions.

Nowadays, the molecular and immunoassay methods are widely used to detect COVID-19 deploying virus RNA proteins; however, they are not useful for POC applications due to limitations, such as the need for expensive equipment and experienced personnel, as well as the high cost of diagnostic services. Thus, there is a need for new diagnostic strategies, and improvement of the diagnostic methods is required due to the global outbreak of COVID-19. An optical biosensor is a very critical component of POC devices because they usually estimate the level of biological markers or any chemical reaction by generating signals that are related to the concentration of the analyte, the high sensitivity of these systems makes it possible for early diagnose of disease [114,115]. SERS is a commercializable, non-invasive spectroscopic, and label-free diagnostic technique that enables the detection of individual molecules in high resolution. High sensitivity, the sharpness of the Raman signals, and narrow bandwidth in SERS are comparable to fluorescent labels such as quantum dots with broad absorption/emission bands [116,117]. The properties of the narrow bandwidth of SERS show distinctive molecular information even at the level of chemical moieties, permitting the detection of multiple without being limited by spectral overlapping [118].

Chemical interactions between analytes and substrate and magnetic field enhancement are effective in increasing the intensity of the Raman signal from 10^4^ to 10^14^ [119]. The SERS largely depends on the type of substrate and its preparation. SERS platform can be designed in the form of colloidal metal solutions as well as thin precipitated or nanometer films, and the most common types of SERS-active substrates showing the largest effects are colloidal silver and gold, and evaporated films of these metals [120,121,122]. In a similar study, Yeh et al. showed that by using the synthesized nitrogen modified carbon nanotubes decorated with Au NPs, virus detection would be very accurate in a short time. In addition, the detection could be obtained with 10^2^ EID_50_/mL (50% egg infective dose per microliter), with a virus specificity of 90%. After that, they demonstrated by using the SERS-based techniques, the abundance of viral-specific reads significantly increased from 4.1 to 31.8% for parainfluenza and from 0.08 to 0.44% for the influenza virus. This enrichment method, coupled with Raman techniques, constitutes an innovative system that could be used to quickly track and monitor viral outbreaks in real-time [123]. Furthermore, Zhuang et al. reviewed different point-of-care techniques in the assistance of analytical chemistry with the emphasis on SERS-based technologies and represents that by using different polymer-based membranes in the microfluidic devices, these nanosystems can be easily optimized to detect different types of viruses [124].

Functionalized Magnetic NPs (MNPs) can be used as a potential candidate to extract viral RNA. Isolation of the virus RNA from several samples within 20 min by a rapid method via performing lysis and elution, all in one step, is one of the advantages of these NPs [125]. The elimination of elution step in the RT-PCR technique using -RNA MNPs, offers a reduction in time as well as a risk of contamination in the diagnosis of COVID-19.

Suspended MNPs used in RNA extraction, and MNPs-based thin films have been suggested as potential substrates in SERS-based diagnostic techniques for COVID-19 RNA. In this assay, after binding the RNA virus to the substrate, the detection of the virus is possible by analyzing the Raman signals from the MNPs-substrate interaction with RNA viruses that can help develop diagnostic POC systems.

Obviously, each technique has pros and cons, and SERS is no exception. Some of the most important disadvantages of SERS include the need for a homogenous sample, the substrate destruction over time that reduces the signal, as well as the ability to repeat SERS substrate signal in a substrate [126]. However, various strategies have been reported to increase the stability and inhibition of SERS substrate oxidation, such as covering the substrate surface with thin layers of SiO_2_ [127], and MnO_2_ [128], etc., or using graphene-based metallic nanostructure [129]. However, despite the limitations of SERS, it is still an attractive method to identify a variety of analytes and even known and unknown viruses due to its high sensitivity and the unique characteristics of its spectra.

## 4. Nanotechnology to Detect SARS-CoV-2

Today, many research groups are working on diagnosing the Coronavirus, and several techniques have been proposed based on analytical chemistry principles. The great competition among scientists on the treatment and detection of this virus has had amazing results; however, the routine techniques require special laboratory facilities. More importantly, diagnosing the virus outside the human body and any living organisms can help mass prevent infection. In this regard, nanotechnology can help scientists to detect the virus outside the living organisms with high accuracy and selectivity. The purpose of this review article is to have a literature survey, with the purpose of providing a logical and scientific solution for detecting the virus at different levels using optical techniques. In this regard, the use of porous nanostructures that are capable of absorbing/adsorbing the virus is very important. For example, scientists can use different Au or Ag NPs and optimize them based on their size and surface functional groups. In the next step, the color of these NPs or modified nanostructures change as per the presence of a virus, or even the concentration of the virus in the air can be measured using different color spectra and fingerprint techniques [130,131,132,133]. It should be noted that the technique proposed in this article is based on the optical properties of doping the NPs in porous nanostructures that have been coated on the surface of the mask and clothes.

### 4.1. Based on Gold NPs

Au NPs can be used as detection probes in virus surveillance because of their optical/electrical features [134]; they can have diverse morphology such as cubes, rods, prisms, tetrapods, shells and hollow structures. Since they have free electrons that called “plasmons,” exerting light will push the plasmons to move far from the atomic core, which causes a phenomenon called surface plasmon resonance (SPR). Therefore, enhancing or quenching effects of SPR in Au NPs is due to their interaction with a guest material and can be able to apply as an indicator for biosensor applications [135]. The first phenomenon related to Au NPs for viral detection is resonance light scattering. For example, an SPR method is designed which relied on hollow nanostructures modified with Au NPs and a DNA 3-way junction to detect label-free H5N1 avian influenza virus with a limit of detection (LOD) of 1 pM [136]. It should be noted that Raman spectroscopy and dynamic light scattering are other methods to detect resonance light scattering [135]. Furthermore, colorimetric alternation is the second group of viral detection techniques based on Au NPs. As an example, Wang et al. conducted research for visual detection of hepatitis B virus (HBV) and hepatitis C virus (HCV) with an Au NPs-supported gene probe that relied on capturing oligonucleotides tethered on a glass surface with using a complementary DNA (cDNA) target. This study performed a sandwich hybridization/nano Au amplification/Ag-staining system based on using colorimetric detection of two probes to monitor HBV and HCV [137]. In similar research, a two-hybridization gene detection chip (a form of microarray technology) was proposed, which contained an initial hybridization between the PCR amplified HBV gene and the capturing probe on the chip. The second hybridization was built between the HBV gene and a probe capped on Au NPs [138]. Finally, the third option for Au NPs-based viral detection is relying on fluorescence quenching or enhancing. A biosensor designed based on fluorescence resonance energy transfer (FRET) system, including Au NPs and FAM, to detect the HBV DNA sequences. By adding FAM-tagged ssDNA into Au NPs suspension, they get adsorbed on the positive surface of Au NPs (previously charged positively with cetyltrimethylammonium bromide). So, a FRET process was observed from FAM to Au NPs and FAM fluorescence intensity quenched. Adding cDNA to the complex solution decreased the fluorescence intensity even more, and the detection limit was as low as 15 pM [139]. In order to design an efficient probe to detect the Zika virus, a localized surface plasmon resonance (LSPR) system was used with signals from plasmonic NPs to mediate the fluorescence signal originating from quantum dots nanocrystals in a molecular beacon probe. This study compared four different plasmonic NPs, including Ag, Au, Core/Shell Au/Ag, and alloyed AuAg NPs that were functionalized with 3-mercaptopropionic acid; these plasmonic NPs were separately conjugated with a capped CdSeS quantum dot. The basis of this system is using LSPR from a plasmonic NPs to mediate fluorescence signal to quantum dots, which was triggered as a result of hybridization between the Zika virus and DNA loop sequence of molecular beacon probe [140].

### 4.2. Based on Ag NPs

The optical properties of silver (Ag) NPs are similar to those of Au NPs. Silver NPs have been widely used in the SERS system in combination with different metal nanoarrays, which can be able to improve the plasmonic activity. Essentially, by using plasmonic techniques in assistance of Raman labeling with active components and applying electromagnetic field, different viruses such as HBV can be able to detect with a detection limit of 50aM [141]. Moreover, a fluorescence enhancement strategy based on Ag NPs functionalized with quantum dots (Cy-3-probe) and hybrid probes to detect HBV DNA sequences with the LOD of 50fM are considered as a promising approach, the LOD of that is 150-fold lower than the un-enhanced fluorescent technique [142].

### 4.3. Based on Magnetic NPs

Magnetic bead-based immunoassay is a technique centered on attaching antibodies to a magnetic bead, then separating the target from the sample by exerting a magnetic field. This process can be completely designed to ensure on a chip. For example, MnFe_2_O_4_ magnetic beads conjugated with influenza “A” antibody was produced to detect the target viruses. The final detection was performed visually based on fluorescence intensity by a fluorescence microscope evaluating R-phycoerythrin as fluorescence-dye; LOD described was as low as 0.007 hemagglutination units [143]. Moreover, a sandwich immunoassay based on magnetic beads introduced electrochemically can detect the IgG antibodies against HBV antigens. In this method, magnetic beads serve as bioreaction platforms while Au NPs are electroactive labels with the limit of detection equal to 3 mIU of the target antibody (International Unit) per mL of human serum sample [144]. Another magnetic-based immunoassay was applied to monitor nucleoprotein molecules of the H1N1 virus by magnetization response spectroscopy. The magnetic particles comprising iron oxide NPs and IgG polyclonal antibodies were attached on the surface of magnetic particles to produce a cross-linking between magnetic particles and nucleoprotein molecules of the H1N1 virus; LOD reported to be 44 nM in this case [145].

### 4.4. Based on Metal-Organic Framework

Based on the above-mentioned discussions, porous nanomaterials can be used for the detection of different pathogens. In this regard, the analyte, pathogen, does not need to be absorbed by the porous nanomaterials; however, the pathogen just needed to interact with the surface of the MOF that is modified by different NPs. By this interaction, different Off–On or On–Off optical mechanisms can be optimized to detect the pathogen, and in this case, different optical active components can be used as quenchers or activators [31,146,147]. For instance, a Cu-based MOF was synthesized to simultaneously detect two distinctive fluorophore-labeled DNA probes for fluorescent identifying of Dengue and Zika RNA sequences. The detection limit for synchronous fluorescence analysis reported to be 184 and 121 pM, respectively. This Cu-based MOF has an affinity toward carboxyfluorescein (FAM), and 5(6)-carboxyrhodamine, triethylammonium salt-tagged single-stranded probe DNA and the study reported no cross-reaction between the two probes during synchronous detection [148]. Additionally, a Cu-based MOF relying on its affinity for 5’6-FAM (as a fluorescent DNA probe) was designed to detect the H1N1 antibody in a serum sample virus with a detection limit of 1.6 nM [149]. In another case, a three-dimensional MOF was deployed to detect HIV-1 double-stranded DNA based on its interaction with FAM DNA probes [150]. However, in the case of SARS-CoV-2, there is no need to detect the exact genetic material and genetic sequence on the surface of mask or even clothes due to the considerable differences between the concentrations of the SARS-CoV-2 with others. To be exact, by using a fingerprint fluorescence pattern, which has been optimized before, the exact range of concentration of SARS-CoV-2 on the contact surface of the gas and solid phases can be measured by optical changes. Moreover, if the MOF based biosensors successfully work for HIV-1, H1N1, ZIKA, and other pathogens detections with significant accuracy and LOD, then the morphology and optical-based biosensor for detection of SARS-CoV-2 should work as well.

**Figure 7:**
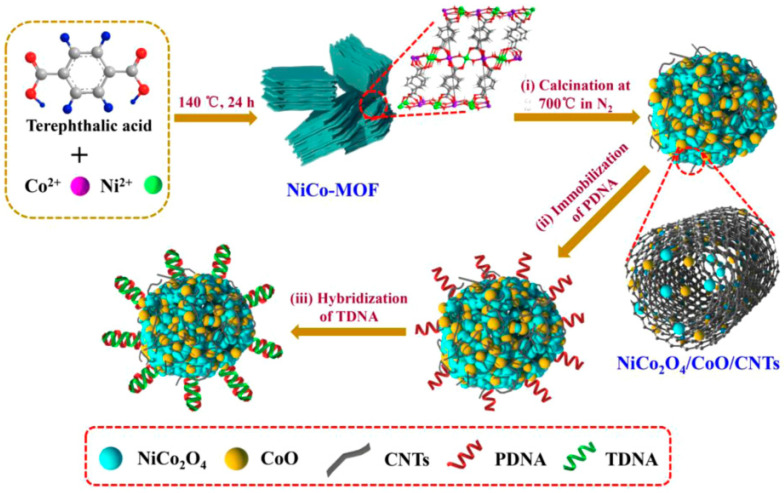
Schematic depiction of various steps to prepare NiCo_2_O_4_/CoO/CNTs-based assay to identify HIV-1 DNA; producing NiCo_2_O_4_/CoO/CNTs-based assay, The DNA probes immobilized, and the detection of the HIV-1 DNA [151]. It is reprinted with permission from Elsevier.

However, there are other classes of MOFs composites which perform as a size-selective filter, followed by SERS to detect the low change of concentrations [34]. A bimetallic NiCo-based MOF was synthesized for immobilizing the DNA probe for HIV-1 and electrochemically detecting the virus, as illustrated in Figure 7 [151]. The limit of detection was as low as 16.7 fM toward HIV-1 DNA. In addition, A chromium-benzenedicarboxylates (MIL-101) MOF was applied to monitor respiratory syncytial virus (RSV) gene sequences in a label-free technique relying on fluorescence anisotropy [152,153,154,155].

## 5. Conclusion and Future Outlook

A very important and often overlooked aspect that has not been addressed in this field and that can revolutionize the detection and prevention of the virus is its detection on masks or special clothes using optical techniques. Such a strategy can easily detect carriers and greatly prevent people with a low immune system from being infected by the virus. With that objective in mind, different coating layers can be used on the surface by using MOFs that have active metal NPs such as Au NPs and which in the presence of the SARS-CoV-2, changes the color of the NPs considerably, namely from red to blue, thus detecting even trace concentrations of the SARS-CoV-2. This hypothesis is based on recent research that reveals the sustenance of the SARS-CoV-2 in the air for more than 3 h and its movement on the aerosol particles up to 8 m. MOFs with different range of porosity, various sizes, and interconnected volumes can be synthesized. All of them have the ability to interact on the surface with cysteine-sensitive molecules, the active component of the SARS-CoV-2 proteins that are being targeted, as well as loading of different metals with active optical properties.

As the COVID19 pandemic is exponentially spreading and affecting millions of innocent people around the world, this article aims to stimulate researchers to think creatively to generate new nanosystems for the prevention, diagnosis, and treatment, and significantly reduce the spread of the disease. To deal with such globally damaging virus, a well-coordinated, timely, fast, and effective response is the essence of mitigation efforts by humans.

## Figures and Tables

**Table 1:** Sensitive of detection, advantages, and limitations of current methods in diagnosis of COVID-19.

Methods	Sensitivity of Detection	Analyses of Time	Advantages	Limitation	Ref
Culture	30–50%	1–3 days	Appropriated for slower-growing pathogens	Risk of contamination, time-consuming	[46]
Next-generation sequencing (NGS)	-	Around 1–2 weeks	Ability to fully recognize the genome, even mutations. Detection based on genes	Time-consuming, short reads, need for technical expertise	[67,68]
Immunoassays Methods (e.g., ELISA)	20–80%	About 2 h with the kit	High sensitivity, ability to detect IgG and IgM antibodies in serum. Detection based on antibodies	Expensive to prepare antibody, limit the amount of antigen in samples, antibody instability	[69]
RT-PCR	95%	2 h	Highly sensitive method	Requires expensive instruments, long reaction times	[22,55]
LAMP	>95%	About 30 min	Rapid, simple, high specificity and specificity	Difficult in primer design, false-positive results	[62,70]
Computed tomography (CT)	97%	Rapid	Rapid analysis	Non-specific	[8]
